# Implementation Strategies to Improve Engagement With a Multi-Institutional Patient Portal: Multimethod Study

**DOI:** 10.2196/28924

**Published:** 2021-10-28

**Authors:** Jamie Keiko Fujioka, Julia Bickford, Jennifer Gritke, Vess Stamenova, Trevor Jamieson, R Sacha Bhatia, Laura Desveaux

**Affiliations:** 1 Women's College Hospital Institute for Health Systems Solutions and Virtual Care Toronto, ON Canada; 2 Northern Health Authority Prince George, BC Canada; 3 London Health Sciences Centre London, ON Canada; 4 Unity Health Toronto Toronto, ON Canada; 5 Department of Medicine University of Toronto Toronto, ON Canada; 6 Institute of Health Policy, Management and Evaluation University of Toronto Toronto, ON Canada

**Keywords:** patient portal, electronic health record, patient health record, digital health

## Abstract

**Background:**

Comprehensive multi-institutional patient portals that provide patients with web-based access to their data from across the health system have been shown to improve the provision of patient-centered and integrated care. However, several factors hinder the implementation of these portals. Although barriers and facilitators to patient portal adoption are well documented, there is a dearth of evidence examining how to effectively implement multi-institutional patient portals that transcend traditional boundaries and disparate systems.

**Objective:**

This study aims to explore how the implementation approach of a multi-institutional patient portal impacted the adoption and use of the technology and to identify the lessons learned to guide the implementation of similar patient portal models.

**Methods:**

This multimethod study included an analysis of quantitative and qualitative data collected during an evaluation of the multi-institutional MyChart patient portal that was deployed in Southwestern Ontario, Canada. Descriptive statistics were performed to understand the use patterns during the first 15 months of implementation (between August 2018 and October 2019). In addition, 42 qualitative semistructured interviews were conducted with 18 administrative stakeholders, 16 patients, 7 health care providers, and 1 informal caregiver to understand how the implementation approach influenced user experiences and to identify strategies for improvement. Qualitative data were analyzed using an inductive thematic analysis approach.

**Results:**

Between August 2018 and October 2019, 15,271 registration emails were sent, with 67.01% (10,233/15,271) registered for an account across 38 health care sites. The median number of patients registered per site was 19, with considerable variation (range 1-2114). Of the total number of sites, 55% (21/38) had ≤30 registered patients, whereas only 2 sites had over 1000 registered patients. Interview participants perceived that the patient experience of the portal would have been improved by enhancing the data comprehensiveness of the technology. They also attributed the lack of enrollment to the absence of a broad rollout and marketing strategy across sites. Participants emphasized that provider engagement, change management support, and senior leadership endorsement were central to fostering uptake. Finally, many stated that regional alignment and policy support should have been sought to streamline implementation efforts across participating sites.

**Conclusions:**

Without proper management and planning, multi-institutional portals can suffer from minimal adoption. Data comprehensiveness is the foundational component of these portals and requires aligned policies and a key base of technology infrastructure across all participating sites. It is important to look beyond the category of the technology (ie, patient portal) and consider its functionality (eg, data aggregation, appointment scheduling, messaging) to ensure that it aligns with the underlying strategic priorities of the deployment. It is also critical to establish a clear vision and ensure buy-ins from organizational leadership and health care providers to support a cultural shift that will enable a meaningful and widespread engagement.

## Introduction

### Background

Effective health care requires patient-centered and integrated health services coordinated across multidisciplinary teams and delivery settings [1,2].

Efficient communication and health information exchange between patients, providers, and caregivers across settings is currently lacking, jeopardizing patient safety and health system costs [3-5]. To address this gap, health systems are increasingly investing in patient portals that enable timely and comprehensive information access through a single channel by patients [6,7]. Broadly speaking, patient portals offer secure electronic access to clinical information collected by one or more health care institutions [8,9]. They are related to but distinct from personal health records, which store health data compiled, managed, and updated by patients [10].

Comprehensive patient portals that enable access to data from multiple health services (ie, internal and external laboratories, diagnostic images, and discharge reports) have been shown to reduce duplicate testing [11] and enhance communication [12,13]. The primary argument for patient portals is not that they intend to create value for money; rather, they foster patient empowerment and self-management and improve patients’ engagement in their care [14]. The downstream anticipated impact of this includes improved health outcomes [12,13,15-18] and reduced health resource usage [19-22]. However, suboptimal implementation threatens the potential realization of benefits. For instance, approximately 90% of the US health care systems and providers offer patient portals; however, only 15% to 30% of patients report using these resources [23,24]. Australia has noted similar adoption rates, with only 22% of citizens registered on their national portal since its launch in 2012 [25]. Several factors hinder adoption and use, including privacy concerns [18], low motivation to enroll [26], design and usability problems [18,27,28], and issues related to health or technical literacy [28] and equitable access [29]. In addition, the implementation of a comprehensive multi-institutional portal requires system interoperability and interorganizational coordination, which is often difficult to achieve across fragmented health services [7,15].

Although barriers and facilitators to patient portal adoption are well documented [9,14,17,30], there is a dearth of evidence on how the implementation process interacts with individual and organizational contexts [13]. This makes it difficult to discern the specific processes or factors that underpin the success or failure of the implementation. In response to this knowledge gap, we conducted an evaluation of a multi-institutional patient portal deployment to understand the experiences of patients, health care providers, and administrators.

### Objectives

The specific objectives are to explore how the implementation approach impacts the adoption and usage of a patient portal and to identify lessons learned when implementing multi-institutional patient portals that transcend traditional boundaries and disparate systems.

## Methods

### Study Design

This multimethod study included an analysis of routinely collected usage data that were extracted from the portal by the vendor to understand use patterns during the first 15 months of implementation (between August 2018 and October 2019). In addition, qualitative semistructured interviews were conducted with patients (and their informal caregivers when applicable), health care providers, and administrative stakeholders to understand how the implementation approach influenced patient and provider experiences and overall usage. Ethics approval was received from the Research Ethics Board of the Women’s College Hospital (REB approval no. 2019-0035-E). The reporting of this study was guided by the Consolidated Criteria for Reporting Qualitative Research checklist [31].

### Study Setting

Southwestern Ontario (SWO) is a region of Ontario, Canada, that includes rural, suburban, and urban populations. The approximately 3.6 million residents of SWO represent 30% of Ontario’s population [32]. Over 45,000 health care providers in SWO securely access publicly stored patient information through a regional webportal (ClinicalConnect) [32]. ClinicalConnect is a provider-facing viewer that consolidates data from 72 acute care hospitals, 4 home and community care organizations, and 4 regional cancer care programs in SWO [32]. It enables access to 4 provincial data repositories that house diagnostic imaging reports, drug information, laboratory results, and acute care information.

In 2017, Hamilton Health Sciences (HHS), a hospital network consisting of 7 hospitals and a regional cancer center, received funding from Canada Health Infoway, a not-for-profit organization funded by the federal government, to deploy a multi-institutional patient portal for residents in the region [33]. This initiative represents one of the largest deployments of a patient-facing digital health access channel in Ontario. To develop the portal, HHS collaborated with Sunnybrook Health Sciences Centre, a Toronto hospital that adapted its hospital-developed patient portal (MyChart) for the SWO region. To enable access to information across multiple systems, MyChart was integrated with ClinicalConnect rather than being directly tethered to individual institutional electronic patient record systems. Organizations in SWO contributing data to ClinicalConnect were required to sign agreements to enable their data to flow to MyChart for patient access.

The MyChart implementation was launched in August 2018, with a target of 65,000 registered users by December 2019. MyChart was rolled out in stages, as site agreements were finalized with the participating organizations. Patient enrollment was only available in person at each site at the time of this study but was later made available via the internet in March 2020 because of the COVID-19 pandemic. Participating sites were responsible for independently determining what data would be uploaded and with how much delay and for creating implementation processes, such as onboarding of the patient and provider to the portal. Participating sites also had the flexibility to control the availability of portal features (eg, direct messaging) for patients accessing care at their sites (a full list of functionalities is given in Textbox 1). As of January 20, 2020, there were 48 sites contributing data to the regional MyChart and 38 sites actively offering the portal to their patients out of a possible 57 sites that had signed data-sharing agreements in SWO.

**Key functions of the regional MyChart patient portal.**

**Regional MyChart patient portal functionality**

- Access a subset of their clinical information from any location at any time.
- Record and manage certain personal health information electronically.
- Delegate viewing of their record to caregivers and providers who accept a patient’s request to receive access through their own MyChart accounts.
- Send direct messages to authorize MyChart account delegates (ie, clinicians), although very few organizations leveraged this feature.

### Participant Recruitment

#### Overview

Initial recruitment used purposive and snowball sampling of providers and administrative staff at 2 early adopter organizations—HHS and Huron Perth Healthcare Alliance. Patients were recruited through convenience sampling, and recruitment posters were posted in waiting rooms and hospital staff notified eligible patients of the study. We used a maximum variation sampling strategy with the aim of recruiting a representative patient and provider sample. In particular, age, sex, health care condition, and geographic location (urban or rural) were considered. Provider recruitment targeted different health care professions and diverse clinical areas. Interviews were also sought with administrative stakeholders representing a variety of organizational roles and responsibilities in relation to implementation. Owing to slower than anticipated adoption of the portal, recruitment was expanded beyond HHS and Huron Perth Healthcare Alliance to include all partner organizations in SWO. Interview participants were asked to refer colleagues or contacts that could provide relevant insights following the completion of their interview.

#### Data Collection

Sunnybrook Health Sciences Centre provided aggregate, deidentified use data between August 2018, when MyChart was first implemented, and October 2019, the most recent month at the time of analysis. Data elements included the number of registered users, total number of log-ins per user, number of users who granted delegate access, number of users who recorded personal health information independently, and the page views for the various sections. In addition, they provided a summary of the number of users who logged in more than once and more than five times during October 2019.

Semistructured qualitative interviews, which lasted 30 to 60 minutes, were conducted over telephone between April and December 2019 with administrative implementation stakeholders, patients, caregivers, and health care providers who were informed of the study objectives. The interview guides included questions on implementation strategy, barriers and enablers to patient portal use, impact on the Quadruple Aim framework (patient experience, provider experience, health care costs, and health outcomes) [34], and suggestions for improvement. Interviews were conducted by members of the research team (JB and JG) who had no relationship with eligible providers or patient or caregiver participants. Some of the administrative stakeholders were familiar with the interviewers given their roles in the SWO community. All interviews were audio recorded and transcribed verbatim by a third party. Verbal consent was obtained before the interviews, and participants were given a US $13 gift card as a token of appreciation.

### Data Analysis

Aggregate quantitative data were analyzed using descriptive summary statistics (eg, calculating frequencies and averages) with Excel software (Microsoft Corporation) to understand use patterns, including which features of the portal were most frequently used and how often the portal was accessed.

Qualitative data were analyzed using an inductive thematic analysis approach, which aimed to identify recurring patterns across a data set [35]. Two researchers (JB and JG) developed a preliminary codebook by independently coding the first 3 interview transcripts. The 2 researchers then discussed the codes, clarified and resolved discrepancies, and created a project codebook. The codebook was then discussed with the research team to ensure alignment with the study objectives and applied to additional transcripts as interviews were completed. Throughout this process, JB and JG met periodically to iteratively refine the codebook to reflect new codes, merge related codes, and resolve discrepancies. Once coding was complete, codes were synthesized into preliminary themes that were mapped back to the study objectives. Refinements and specifications of thematic categories and subcategories and relationships between themes were determined through in-depth discussion and negotiated consensus between members of the research team (JB, JG, JKF, and LD). NVivo 12 software (QSR International) was used to assist with coding and analysis.

## Results

### Quantitative Use Data Findings

Between August 2018 and October 2019, a total of 15,271 registration emails were sent, with 67.01% (10,233/15,271) of patients registered for an account across 38 sites. The median number of patients registered per site was 19, with considerable variation (range 1-2114). Of the total number of sites, 55% had less than or equal to 30 registered patients (21/38), whereas only 2 sites had over 1000 registered patients. Among the registered patients, 92.00% (9,414/10,233) logged in at least once during the 15-month evaluation period. At the time of this study (October 2019), only 23.69% (2,424/10,233) had logged in more than once during the preceding month, with less than 3.83% (392/10,233) accessing the portal more than five times. High-traffic information pages included radiology exams and laboratories, discharge summaries, pathology results, and medications (Table 1). Only 2.98% (305/10,233) of registered users appointed a delegate, and less than 1% (73/10,233) actively shared information with their providers. A small number of patients (140/10,233, 1.37%,) independently recorded data in their patient health records via the portal.

**Table 1:** Top information page views accessed between August 2018 and October 2019—views of the lists associated with each category.

Category	Page views (August 2018 to October 2019)
Radiology	46,268
Laboratory results	43,799
Discharge summaries	18,377
Pathology results	16,884
Medications	14,689
Allergies	13,486
Microbiology results	11,771
Record summary	11,311
Home and community care	8507
Blood bank tests	8223

### Qualitative Data Findings

A total of 42 individual interviews were conducted with stakeholders from 17 organizations within the SWO region to understand how the implementation unfolded and the factors underlying the adoption rates. The majority of participants (11/42, 26%) were recruited from HHS, followed by the Heron Perth Health Alliance (7/42, 17%) and the London Health Sciences Centre (5/42, 12%). The interview participants included 18 administrative stakeholders involved in the MyChart implementation, 16 patients, 7 health care providers, and 1 informal caregiver (Table 2). Administrative stakeholders included individuals involved in clinical leadership, clerical roles, patient experience teams, and information technology (IT). Among the patients and caregiver interviewed, 76.5% (13/17) had used MyChart, 11.8% (2/17) had registered but had not accessed it because of log-in challenges, and 11.8% (2/17) did not have access because of challenges with registration. The average age of patient or caregiver participants was 54 years, and all indicated that they were managing a chronic illness.

**Table 2:** Characteristics of the interview participants (N=42).

Interview participants				Participants, n (%)
Administrative stakeholders, (n=42)				18 (43)
**Patients or caregivers,** **(n=42)**				17 (40)
	Patients (n=17)			16 (94)
	Caregivers (n=17)			1 (6)
	**Gender (n=17)**			
		Female	11 (65)	
		Male	6 (35)	
**Registered MyChart user (n=17)**				
	Yes—have registered log-in and used it			13 (76)
	Yes—have registered but have not used it			2 (12)
	No			2 (12)
**Geographic area**				
	Urban (n=32)			8 (47)
	Rural (n=17)			4 (23)
	Suburban (n=17)			3 (18)
	Small town (n=17)			2 (12)
**Education (n=17)**				
	Bachelor’s degree			77 (41)
	College diploma or certificate			6 (35)
	Some college			2 (12)
	Postgraduate education			1 (6)
	High school			1 (6)
**Providers (n=42)**				7 (17)
	**Health profession (n=7)**			
		Physicians	4 (57)	
		Nurse or nurse practitioner	2 (29)	
		Nonnursing allied health professional	1 (14)	

Five key themes emerged. They described how the implementation process influenced adoption rates and lessons learned that can be leveraged to increase the uptake of similar patient portal models.

#### Optimize the Patient Experience by Prioritizing Data Comprehensiveness

All interview participants unanimously endorsed the importance of leveraging technology to enable access to a comprehensive patient record, which they believed would reduce health system fragmentation by streamlining access to information. Patients perceived the benefits of this to include timely, efficient, and remote access to their health information, which enabled them to feel more prepared to manage their health concerns. Generally, patients had positive perceptions of MyChart because of its user-friendliness and ability to provide them access to pertinent health information (eg, test results, radiology reports). In addition, they felt that their relationships with their providers could be improved because the patient portal fostered increased transparency, knowledge, and empowerment. However, several data gaps were identified that limited perceived value, such as details related to home and community care (eg, care plans) and diagnostic images (not just reports). Particular emphasis was placed on the value of accessing clinic notes:

What’s missing are the clinical notes...So, you’re sitting with the physician say prior to chemo, that he’s reviewed A, B, C with you. You know, and sometimes, and especially if it’s chemo, there is an unfortunate thing called chemo brain or chemo fog and you think you’ve asked questions, but you know, three days later you’re like, did I ask that? So, to go back and double check that I indeed did ask that question [helps me make sure] I don’t want to waste the physician’s time [by following up].

Participant 40, Patient

Participants suggested that data comprehensiveness could be further improved by expanding the scope of the regional data to include information currently unavailable in MyChart but available in other provincial repositories, such as certain drug and immunization records.

In addition, educational materials (eg, information on how to interpret laboratory results) were perceived to be valuable in helping patients when interpreting clinical information and would likely improve engagement with the portal. Administrative stakeholders emphasized that core elements that enable access to comprehensive information, such as interoperability and integration with electronic medical records, should be prioritized during implementation. This aligns with patient motivations for engaging with the portal and its overall objective as a service within the system. Although extra features (ie, secure messaging with providers and appointment reminders) that require additional workflows and provider engagement were desired by patients, administrative stakeholders perceived these should be considered based on local needs and implemented in a graduated manner to keep the implementation in scope and logistically feasible.

#### Enhance Adoption by Using a Broad Rollout Strategy Instead of Targeting Specific Departments

Increasing patient awareness of the portal and implementing a clear and simple registration process with the aid of hands-on support were identified as adoption facilitators across participant groups. Patients generally perceived the registration and onboarding process to be easy to follow because this was facilitated through the aid of registration clerks. The low uptake in this study was attributed to inadequate marketing and promotion. Many sites used a targeted rollout strategy in select clinical areas instead of an organization-wide approach, which failed to harness broader marketing strategies and ultimately led to patient confusion. This approach was also highlighted as a potential threat to achieving health equity:

I know that some hospitals choose just kind of departments or areas to kind of trial it out. I really don’t recommend that approach. We thought about it and I just said you know what, it has to be, for any success, we’re gonna have to do a big bang theory and do it at all points of registration. So, that would be my recommendation even for large sites. I know it seems daunting, but I think the success is far outweighs for the patient because I don’t think it’s fair when a patient goes to say chemo and they don’t have access to their chart, but the next time they go to DI [diagnostic imaging], they’re asked about MyChart.

Participant 13, Administrative Stakeholder

Most administrative and provider participants felt that a broad rollout strategy would have been more effective, with sites that implemented this strategy describing more effective advertising and communication efforts. In addition, participants across stakeholder groups suggested leveraging diverse patient registration approaches to improve uptake, such as a combination of onsite and web-based enrollment options.

#### Providers Should Be Engaged to Understand and Mitigate Concerns

Gaining clinical buy-ins and alleviating provider concerns around open access to health information was a common challenge acknowledged across participant groups. Providers were specifically resistant to enabling access to their clinical notes because of potential misinterpretation by patients and liability concerns. In contrast, patients expressed a desire to have open access to all their health information. A related tension arose between the patient’s desire for real-time access to information and provider preferences for upload delays to allow time for review and patient communication (ie, when information contained a new diagnosis). Many providers anticipated an increased workload as they expected they would need to assist with patient onboarding, help patients interpret medical jargon and resolve patient misinterpretation of their clinical information. However, these concerns were not realized during the study period. To foster provider acceptance, administrative stakeholders suggested that the patient portal should be framed as a patient service with the clear objective of promoting patient-centered care instead of as a clinical tool. Additional enablers to provider endorsement of the technology included clear, upfront communication about its purpose and visible clinical champions who could allay concerns among peers. In the event that additional features were activated (ie, secure messaging), administrative and provider participants stated that additional efforts should be made to engage providers to consider whether and how to align them with existing workflows:

So when we’re trying to get their [providers’] attention, we’re trying to explain to them that this is a patient service, not a clinical project...it’s something you’re going to offer your patients...and you don’t have to do anything else.

Participant 1, Administrative Stakeholder

#### Change Management Support and Senior Leadership Endorsement Is Central to Early Success

The implementation of MyChart required additional administrative and logistical activities to recruit and onboard multiple sites. Interviews with administrative stakeholders revealed that organization size influenced deployment, with smaller hospitals reporting greater success in rolling out the portal and encouraging adoption than larger organizations. Competing priorities and resource constraints introduced implementation challenges in larger hospitals, which were often attributed to the upfront time and resources required to develop an operational model (ie, developing privacy and security agreements, identifying appropriate age and criteria of consent, and ensuring organizations have the technical requirements to contribute). As such, adoption was more successful when dedicated and protected resources were available to support upfront change management and implementation requirements:

[T]his is actually transformational change in the way we approach the health care encounter. That it’s not just about rolling out access to a portal. And therefore, you need to have dedicated resources. I’m going to be frank and candid. It’s not the sort of work that can just be done off the side of people’s desks and it’s almost what – it feels like we weren’t resourced properly to foster success.

Participant 14, Provider

Another challenge to MyChart deployment was the need to gather stakeholder buy-in from multiple organizations. Within organizations, administrative stakeholders linked slower implementation to a lack of strong and overt senior leadership support. Conversely, rapid portal uptake, engagement, and onboarding were attributed to visible senior leadership endorsement. This was because of the senior leaders’ ability to encourage high organizational motivation and interest to both contribute to the portal and enroll patients:

The CEO was like, let’s go. They were visibly – like they were popping by our booth a few times a day and I think that just – like the optics, it looks good, it shows that they’re interested. They’re approving of this initiative...every single executive came and signed up first thing and they were excited and they were telling staff about it and prompting staff to get registered.

Participant 20, Administrative Stakeholder

#### Regional Alignment and Policy Supports Are Required to Streamline Implementation Efforts

Administrative stakeholders discussed how reconciling differential IT infrastructure to achieve system interoperability was a major barrier to expanding the portal across organizations. Before implementation, HHS had to work with potential enrollment sites to standardize data (ie, test names and medical terminology) for data filters to work consistently. Administrative stakeholders recommended the application of province-wide data standards to resolve these issues.

In addition, implementation was impeded by the need for organizations to gather consensus on complex policy considerations, such as identifying age and capacity to consent and operationalizing proxy access to delegate users. Although organizations appreciated that they had the autonomy to make these decisions to adapt to local needs, participants recommended the development of provincial guidelines and best practice approaches to inform organizational policies:

It’s a bigger conversation about how we partner with families and we don’t have well established policies or limitations.

Participant 24, Administrative Stakeholder

To foster a cohesive and well-integrated digital health information ecosystem, participants highlighted that the implementation of multi-institutional portals should be considered in tandem with institutional or private third-party offerings. As many hospitals within Ontario have developed their own in-house, institutionally tethered portals, 1 participant stated that there needs to be a clearer provincial strategy for promoting patient access to their health data:

One of the criteria is to basically have shared data with patients and have them as active participants in their health and so MyChart is clearly part of that, but I think we’re going to lose people if everyone has different systems and if they have ten different apps. There needs to be a coordinated push on behalf of the ministry or governing bodies to, not mandate, but strongly suggest less and less alternatives because ultimately...you know, I’ve been to all over Ontario. Ottawa, Toronto, Thunder Bay and I don’t want to have to keep signing up for new systems wherever I am. So, I think we need an Ontario-based system.

Participant 27, Administrative Stakeholder

## Discussion

### Principal Findings

Multi-institutional patient portals, such as MyChart, that collect and house information across organizations within a given region can enable more effective patient management by streamlining communication, access to clinical information, and service coordination [22]. The downstream benefits of such portals include increased patient engagement [14] and a reduction in emergency care and hospital admissions [20,22]. Although randomized controlled trials assessing patient portals are lacking, some randomized controlled trials have shown that patient portals can reduce hospital readmissions [36], reduce office visit rates, and result in greater adherence to treatment in comparison with control groups [16]. Our findings suggest that data comprehensiveness, organization-wide deployment, provider engagement, and senior leadership endorsement are central to achieving these aims.

Despite the high perceived value of multi-institutional patient portals, MyChart was adopted suboptimally. The population in SWO is approximately 3.6 million [32], implying that less than 0.3% of individuals within this region registered to use the portal at the time of this analysis. In comparison, the adoption of patient portals in other jurisdictions is estimated to reach 5% to 10% of the targeted population per year, with uptake in larger scale (ie, national or regional) implementations lagging behind smaller, more targeted deployments [37]. The slow growth of larger scale patient portals is attributed to challenges in obtaining alignment and system interoperability across fragmented health services [7,37]. In the absence of a cohesive regional implementation strategy, participating organizations of MyChart had to independently establish processes for marketing patient enrollment and training, resulting in variations in adoption and possibly heterogeneous patient experience. Further, a lack of dedicated resources, change management support, senior leadership endorsement, and clinical buy-in impeded success in several organizations. Strategies to improve change management processes and facilitate senior leadership and clinician support include clearly articulating the value of the technology, building consensus on key decisions and operational processes built around a strategic vision, and investing in the required infrastructure and resources, such as interoperable systems and staff training, to foster success [38].

Our findings highlight strategies for the successful implementation of patient portals. First, the implementation strategy needs to align with the core objectives of the technology. In this case, the portal should focus on enabling access to comprehensive clinical information as an initial priority, given that this is the core functionality of interest and would provide the most benefit to patients. Other features (ie, direct messaging and appointment booking) can be explored in consultation with end users once a plan for comprehensive data access is established and successfully operationalized. Second, implementation processes should mirror patient experience. A blanketed rollout approach across an entire organization, rather than limiting deployment to certain departments, was perceived to be more conducive to facilitating information access that transcends traditional health system boundaries. Organizations that implemented the portal uniformly across the organization were able to more effectively advertise and communicate to patients about the portal. Conversely, segmented implementation exacerbates fragmented access to information, limits patient and clinician awareness and shared understanding of the purpose of the technology. This can create a disconnect for patients as they interact with different services within and across organizations. It is important to note that introducing functionality across an organization does not necessarily imply a one-size-fits-all approach as some flexibility is required to adapt to specific population needs [38]. For instance, special considerations regarding sharing clinic notes, proxy consent, and age of consent should be made for pediatric, mental health, and geriatric patients because of concerns regarding their capacity and autonomy [39]. However, interviewees raised the important point that these issues were better governed by universal best practices rather than by individual organizational idiosyncrasies.

Patient portals and other digital technologies can create value for organizations and health systems but only if the surrounding sociocultural factors are considered [40]. Effective leadership and clinical endorsement of technology can reduce behavioral resistance to change [41]. Patient portals may also precipitate changing dynamics between patients and providers, which underpins the cultural shift toward patient-centered care. Similar to other studies [42,43], we found that tensions between paternalistic and patient-centered medicine need to be resolved to facilitate widespread portal use. In tandem, individuals must be equipped with the necessary time, resources, and tools to carry out activities required for adoption, such as onsite training, enrollment, and technical support [43].

At the organizational and system levels, identifying and developing the right infrastructure is an essential component of strategic planning [38]. Multi-institutional patient portals require standard policy and technical infrastructure to enable data sharing that is consistent across sites. This includes identifying guidelines and best practices to establish access policies (ie, proxy access, age of consent, and data delays). The lack of basic integration and interoperability across institutional boundaries impedes the data comprehensiveness required for portals to function effectively in line with their goals [7,15,42]. The exponential growth of digital technologies across health systems implies the need for a degree of interoperability in alignment with more integrated health care [40,42].

Future research should focus on effectively describing and evaluating the implementation strategies that surround multi-institutional patient portals (eg, the use of champions, patient and provider training, addressing beliefs, etc) to identify effective strategies for promoting uptake. In addition, further studies should focus on evaluating the effectiveness of patient portals against their stated aims, including increased patient engagement in care, improved knowledge, and improved patient experience.

### Limitations

The generalizability of our findings is limited to the early stages of implementation. Our sample may have been biased toward early adopters of technology or participants with limited user engagement. Most patient and provider participants had limited interactions with the patient portal, and their perspectives may not reflect the experiences that emerge alongside a more mature patient portal model. Despite this, all participants believed in the value of multi-institutional patient portals that offer patients comprehensive access to their health information in contrast to institutionally tethered offerings that do not centralize health information from across the health system. It is important to note that participants with limited user engagement did not endorse the current operationalization in its entirety; rather, they described the features and functions of a future state patient portal that would provide value to patients. Considerable benefits would be gained from evaluating the factors associated with sustained engagement in such a model. Although most interviews were conducted with individuals who had experience with the portal, the level of knowledge of and exposure to the portal varied. Owing to slower-than-anticipated adoption, our recruitment approach did not seek to discern between high and low adopters, as there were few *high* adopters at the time of the study. Further evaluations of MyChart should examine if there are characteristic differences (eg, based on patient population, region, or institution) between high and low users. Perspectives from diverse and often hard-to-reach patients were not included (eg, newcomers to Canada, non-English speakers, individuals experiencing housing insecurity); they may experience barriers to accessing technology. Consequently, further work is needed to engage with these populations and determine the impact on access and patient engagement from an equity perspective. Despite this, our study provides useful strategies to inform implementation planning at organizational and system levels.

### Conclusions

Although multi-institutional patient portals can enable efficient access to clinical information from across the health system, successful implementation can be affected by several factors. Without proper management and planning, portals can suffer from minimal adoption from patients and poor support from providers. Data comprehensiveness is the foundational component of patient portals and requires aligned policies and a key base of IT infrastructure across all participating sites. It is important to look beyond the category of the technology (ie, patient portal) and consider its functionality (eg, data aggregation, appointment scheduling, messaging) to ensure that it aligns with the underlying strategic priorities of the deployment. Finally, it is critical to establish a clear vision and ensure buy-in from organizational leadership and health care providers to support a culture shift that will enable meaningful and widespread engagement.

## Abbreviations

HHS: Hamilton Health Sciences
IT: information technology
SWO: Southwestern Ontario
